# Classification of patients with low back-related leg pain: a systematic review

**DOI:** 10.1186/s12891-016-1074-z

**Published:** 2016-05-23

**Authors:** Siobhán Stynes, Kika Konstantinou, Kate M. Dunn

**Affiliations:** Arthritis Research UK Primary Care Centre, Research Institute for Primary Care and Health Sciences, Keele University, Keele, Staffordshire ST5 5BG UK

**Keywords:** Classification, Back pain, Leg pain, Nerve root involvement, Sciatica, Diagnosis

## Abstract

**Background:**

The identification of clinically relevant subgroups of low back pain (LBP) is considered the number one LBP research priority in primary care. One subgroup of LBP patients are those with back related leg pain. Leg pain frequently accompanies LBP and is associated with increased levels of disability and higher health costs than simple low back pain. Distinguishing between different types of low back-related leg pain (LBLP) is important for clinical management and research applications, but there is currently no clear agreement on how to define and identify LBLP due to nerve root involvement.

The aim of this systematic review was to identify, describe and appraise papers that classify or subgroup populations with LBLP, and summarise how leg pain due to nerve root involvement is described and diagnosed in the various systems.

**Methods:**

The search strategy involved nine electronic databases including Medline and Embase, reference lists of eligible studies and relevant reviews. Selected papers were appraised independently by two reviewers using a standardised scoring tool.

**Results:**

Of 13,358 initial potential eligible citations, 50 relevant papers were identified that reported on 22 classification systems. Papers were grouped according to purpose and criteria of the classification systems. Five themes emerged: (i) clinical features (ii) pathoanatomy (iii) treatment-based approach (iv) screening tools and prediction rules and (v) pain mechanisms. Three of the twenty two systems focused specifically on LBLP populations.

Systems that scored highest following quality appraisal were ones where authors generally included statistical methods to develop their classifications, and supporting work had been published on the systems’ validity, reliability and generalisability. There was lack of consistency in how LBLP due to nerve root involvement was described and diagnosed within the systems.

**Conclusion:**

Numerous classification systems exist that include patients with leg pain, a minority of them focus specifically on distinguishing between different presentations of leg pain. Further work is needed to identify clinically meaningful subgroups of LBLP patients, ideally based on large primary care cohort populations and using recommended methods for classification system development.

**Electronic supplementary material:**

The online version of this article (doi:10.1186/s12891-016-1074-z) contains supplementary material, which is available to authorized users.

## Background

To tackle the global burden of low back pain (LBP) [1], researchers in primary care have highlighted the identification of clinically relevant subgroups of LBP as the number one research priority [2]. Extensive work has been published on classification systems, where researchers and clinicians have attempted to subgroup LBP patients into homogeneous populations with similar characteristics, with the aim of optimising management and improving patient outcomes. One subgroup of LBP patients are those with leg pain related to their back pain. Low back-related leg pain (LBLP) is one of the commonest variations of LBP, with about two thirds of LBP patients presenting with it in primary and secondary care settings [3–5].

Leg pain can be classified as either radicular pain due to spinal nerve root involvement (NRI), or referred (non-specific) pain due to back pain that spreads down the leg from structures such as ligament, joint or disc but not involving a spinal nerve root [6]. Leg pain is considered an obstacle to recovery [7, 8], or a marker of severity [4], and the further the pain radiates down the leg, the greater the likelihood of increased levels of disability and health care use, particularly when associated with evidence of positive neurological findings [9].

The majority of published guidelines on LBP [10] advocate identifying patients with leg pain thought to be due to NRI. Treatment options for NRI may be different from those for non-nerve root pain [11], and appropriate diagnosis may therefore reduce unnecessary tests and interventions and result in timelier directing of appropriate diagnostic and treatment resources [12]. However, the diagnosis of radicular pain in clinical practice can be difficult [3, 13], and clinicians may disagree as to its presence or absence in a patient with LBLP [14, 15].

A range of definitions and terms are used to describe LBLP due to spinal NRI (including sciatica, radicular pain, radiculopathy, radiating pain, disc herniation), and various diagnostic criteria are used clinically and in the literature to define populations of LBLP [16–18]. This hampers effective communication between clinicians, and with patients, and can limit applicability of research findings from prognostic and intervention studies if eligibility criteria vary for the supposedly same subgroup of LBLP patients.

Despite the implications to the patient, and the wider community, of having LBLP, its classification has received limited attention in the literature and guidelines, compared to LBP alone, especially in the primary care setting. A systematic review of the scientific literature was carried out to compile an up-to-date review of proposed classification systems for LBLP. The objectives of the review were to (i) describe the various ways LBLP is classified and the methods used to derive the classification systems (ii) appraise the classification systems using a specific tool and (iii) identify how leg pain due to NRI is described and diagnosed in the various systems.

## Methods

### Search strategy

An electronic search was conducted in July 2013 of MEDLINE, EMBASE, CINAHL, AMED, PEDro, Web of Science, Cochrane library, DARE and HTA. All databases were searched from their inception. No date or language restriction was applied. An updated search was performed in August 2015. The Medline search strategy is shown in Additional file 1. Supplementary search strategies included hand searching reference lists of included full-text papers and relevant systematic reviews, and a PubMed search of first authors of the included classification systems, to identify any additional relevant published work. Authors were contacted if any clarification on their system was needed.

### Study selection

The eligibility criteria for study selection used in this review are summarised in Table 1. Titles were initially screened by one reviewer (SS). When eligibility could not be determined on the basis of the title, abstracts were reviewed. To select full text papers, two reviewers (SS, KK) independently screened titles and abstracts of the remaining citations. Any disagreements were resolved through discussion and consensus. Selected full text papers and additional papers identified in reference lists of the included full text papers were screened by the same two reviewers and final agreement was reached on which papers to be included in the review.

**Table 1:** Study eligibility criteria

Published studies were included if they fulfilled any of the following criteria:
• Developed and described an original classification system for back pain that included adult patients with low back related leg pain (LBLP). Leg pain was defined as pain below the gluteal fold. • Adapted an existing classification system that was designed for or included LBLP patients. • Provided approaches to appraising or validating an existing classification system for LBLP.
Exclusion criteria:
• Studies looking at specific spinal “red flag” conditions such as cauda equina syndrome, tumours or spinal fractures or a specific disease cohort such as diabetes. • Studies that only used expensive or advanced investigations or technology more likely to be feasible for secondary care settings (e.g. electromyography, surgical findings, imaging or expensive kinematic equipment) for classification of patients. • Case studies and case series design studies.

### Data extraction and quality appraisal

The framework used to describe and appraise the identified classification systems was originally developed by Buchbinder et al. [19] and subsequently used in other reviews for classifying LBP populations [20–23]. Data extraction included *purpose* of the study; *method of development* referring to either a judgement based approach or using statistical methods; *domain of interest* referring to patient population and setting; *specific exclusions* for patients; *categories* within the system and whether additional dimensions *(axis)* to the condition were considered (e.g. severity or chronicity of symptoms); *criteria used* to assign patients to categories (e.g. clinical examination); and *training and personnel needed* to perform the classification.

Seven criteria were addressed to appraise the methodological quality of the classification systems; these are described in Table 2. A score of 1 was awarded for meeting a criterion, 0.5 for partially meeting a criterion and 0 for not meeting a criterion or unable to score due to lack of evidence. A total score of 7 could be achieved. To derive the score, any supporting studies (reporting on reliability, construct validity, generalisability) for the classification systems were included. Two reviewers (SS and KK) independently appraised the selected classification systems.

**Table 2:** Criteria used to appraise classification systems (adapted from Buchbinder et al. [19])

Criteria	Description
Purpose	Is the purpose, population and setting clearly specified?
Content validity	Is the domain and all specific exclusions from the domain clearly specified?
	Are all relevant categories included?
	Is the breakdown of categories appropriate, considering the purpose?
	Are the categories mutually exclusive?
	Was the method of development appropriate?
	If multiaxial, are criteria of content validity satisfied for each additional axis?
Face validity	Is the nomenclature used to label the categories satisfactory?
	Are the terms used based upon empirical (directly observable) evidence?
	Are the criteria for determining inclusion into each category clearly specified?
	If yes do these criteria appear reasonable?
	Have the criteria been demonstrated to have reliability or validity?
	Are the definitions of criteria clearly specified?
	If multiaxial are criteria of face validity satisfied for each additional axis?
Feasibility	Is the classification simple to understand?
	Is classification easy to perform?
	Does it rely on clinical examination alone?
	Are special skills, tools and/or training required?
	How long does it take to perform?
Construct validity	Does it discriminate between entities that are thought to be different in a way appropriate for the purpose?
	Does it perform satisfactorily when compared to other classification systems which classify the same domain?
Reliability	Does the classification system provide consistent results when classifying the same conditions?
	Is the intraobserver and interobserver reliability satisfactory?
Generalisability	Has it been used in other studies and/or settings?

## Results

### Search results

**Fig. 1:**
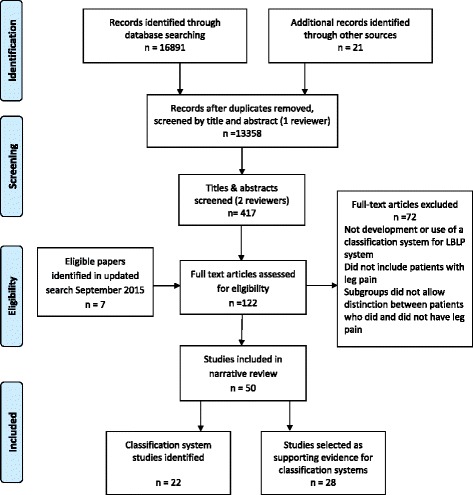
Flow chart of systematic search and study selection

The database search yielded 16,891 references, and an additional 21 papers were identified through hand searches of reference lists. 13,358 records remained after duplicate removal. Following initial screening by one reviewer to exclude papers that were clearly irrelevant, 417 remaining titles and abstracts were selected and subsequently screened independently by two reviewers. 122 articles were identified for full text review. From these, 50 were selected for inclusion which reported on 22 classification systems. A flow diagram summarising the systematic search and study selection process is given in Fig. 1.

### Data extraction and appraisal of selected studies

Based on approaches used in previous LBP classification reviews [22–24], the 22 classification systems were organised into five themes reflecting the purpose and criteria of the classification system: (i) clinical features (ii) pathoanatomical source of pain (iii) treatment based approach (iv) screening tools and clinical prediction rules and (v) pain mechanisms. Data extraction from the papers is presented in Tables 3, 4, 5, 6 and 7. Each table presents one of the classification system themes and gives a descriptive summary of the individual papers within each theme. Quality appraisal of each methodological criterion for the 22 classification systems was done (Additional file 2) and the overall score for each system was calculated (Table 8).

**Table 3:** Data extraction for classification systems: Systems classifying by Clinical Features

Primary author	Purpose	Method of development	Domain of interest	Specific exclusions	Categories	Criteria used	Training/Personnel needed
Barker (1990) [79]	Devise classification meaningful to General Practitioner (GP).	Judgemental approach. GP authorship.	Low Back Pain (LBP). 486 patients attending authors’ GP practice.	Febrile illness, backache accompanied by many other complaints.	1: Acute lumbago 2: Acute mechanical derangement 3: Acute sciatica 4: Sacro-iliac joint (SIJ) 5: Mild sciatica	Patient history, pain location drawings, clinical examination.	None.
Ben Debba et al. (2000) [36]	Assign LBP patients into one of four modified Quebec Task Force Classification categories.	Judgemental and statistical approach. Neurosurgeon authorship.	Persistent LBP. 1,997 patients from tertiary care.	Age under 25, ≥1 prior surgical or interdiscal procedure, no pain in the small of the back.	1: Back pain only 2: Back and above knee pain 3: Back and below knee pain 4: Back and below knee pain with positive straight leg raise (SLR)	Spatial distribution of patient’s pain (from questionnaire). Results of SLR test.	Standardization of SLR performed by clinician or technician.
Glassman et al. (2011) [37]	Develop simple diagnostic classification for use in clinical practice.	Judgement approach. Orthopaedic spine surgeon authorship.	LBP. Case histories compiled.	None.	Clinical Symptoms (relevant to primary care): 1-6: Dominant location of pain 7: Neurogenic claudication 8: Cauda equine Additional axis: Yes Acute/chronic	Patient history and clinical examination.	Not known. Case histories were compiled and reviewed by orthopaedic spine surgeons.
Nachemson and Andersson (1982) [80]	Introduce a simple classification system suitable for use in epidemiological screening.	Judgement approach. Orthopaedic spine surgeon authorship.	LBP.	None.	1: Insufficienta dorsi 2: Lumbago 3: Sciatica 4: Rhizopathy 5: Lumbago sciatica Additional axis: Yes- Duration and recurrence	Patient history and clinical examination. Radiographic results can be used.	Authors report it is simple to use.
Spitzer et al. (1987) [25]	Compile a diagnostic classification system for: clinical decision making; establishing prognosis; evaluating quality of care; Conducting scientific research.	Judgement approach. Multidisciplinary task force representing wide range of disciplines.	LBP.	None.	1: Pain without radiation 2: Pain + radiation proximal extremity 3: Pain + radiation distal extremity 4: Pain + radiation to upper limb/lower limb with neurological signs 5: Presumptive root compression, +ve image 6: Root compression, +ve image 7: Spinal stenosis 8: Post surgical < 6 months 9: Post surgical > 6 months 10: Chronic pain syndrome 11: Other diagnoses Additional Axis: Yes Work and duration	Patient history. Clinical examination and paraclinical test results (laboratory tests, radiography, imaging methods, Electromyography (EMG) nerve blocks).	Able to interpret investigative tests.
Sweetman et al. (1992) [26]	Describe common patterns of LBP and identify clinical tests to help recognize the patterns.	Statistical approach. Rheumatologists authorship.	LBP. 301 patients referred from GP to rheumatology clinic.	Less than 15 or over 75 years old.	1: Persistent unilateral back pain and sciatica 2: Back pain or sciatic switching sides(sacroiliitis) 3: Central/bilateral back pain 4: Lateral flexion or rotation cause pain on the opposite side(facet joint) 5: Back pain at rest on one side but pain on opposite side with several tests (unstable L4/5 syndrome) 6: Dorso lumbar junction conditions 7. Persistent unilateral back pain and sciatica with loss of lower limb reflex (Disc with nerve root compression)	Questionnaire and clinical examination and x-ray.	Uses a computer algorithm for pattern recognition.

**Table 4:** Data extraction for classification systems: Systems classifying by Pathoanatomy

Primary author	Purpose	Method of development	Domain of interest	Specific exclusions	Categories	Criteria used	Training/Personnel needed
Bernard and Kirkaldy Willis (1987) [41]	Determine pathology causing LBP.	Judgement approach. Orthopaedic surgeon authorship.	LBP. Medical record review of 1293 patients, majority of whom had failed initial treatment by primary care physicians.	None.	Group A:well recognized syndromes 1. Herniated nucleus pulposus 2. Lateral spinal stenosis 3. Central spinal stenosis 4. Spondylolisthesis 5. Segmental instability Group B:less well recognized syndromes 6. Sacroiliac joint 7. Posterior joint 8. Maigne’s syndrome 9. Gluteus maximus 10. Gluteus medius 11. Quadratus lumborum 12. Piriformis 13. Hamstring origin 14. Tensor fascia latae Group C: remaining syndromes 15. Pseudarthrosis 16. Non specific 17. Post fusion stenosis 18. Anklyosing spondylitis 19. Disc space infection 20. Tumour 21. Arachnoiditis 22. Lateral femoral nerve entrapment	Medical records and response to treatment which included: manipulation/stretching; injections; radiofrequency denervation; palpation; joint motion tests, neural tension tests and neurological testing, response to surgery, pain provocation palpation, xray and computed tomography (CT) scans.	None.
Cassisi et al. (1993) [40]	Explore differences between two groups of chronic LBP patients.	Judgement approach. Neurosurgeon authorship.	Chronic LBP. 151 patients in tertiary care.	Neoplasm, mechanical, toxic-metabolic, inflammatory-infectious, vascular and psycho-physiological conditions.	Myofascial pain. Disc herniation.	Patient history and clinical examination.	None.
Hahne et al. (2011) [38]	Identify patho-anatomical subgroups with subacute LBP. For use in a randomised controlled trial (RCT): the STOPS trial.	Judgement approach including an expert panel of physiotherapists. Physiotherapy authorship.	LBP +/- leg pain. Subacute pain lasting between 6 weeks and 6 months.	Red flags, recent spinal injections, previous spinal surgery, recent regular physiotherapy treatment.	1: Reducible discogenic pain 2: Non reducible discogenic pain (not responsive to mechanical loading strategies) 3: Disc herniation with associated radiculopathy 4: Facet joint dysfunction 5: Multi-factorial persistent pain	Patient history and clinical examination.	Unclear what specific training is needed for classification.
Paatelma et al. (2009) [44]	Evaluate the reliability of a patho-anatomical classification system.	Judgement approach. Physiotherapy authorship.	LBP +/- leg pain. 21 patients.	Age > 56, LBP > 3 months.	1: Discogenic pain 2: Lumbar instability 3: Spinal Stenosis 4: Segmental dysfunction/facet pain 5: SIJ dysfunction/pain	Patient history and clinical examination.	5 ½ day training sessions to standardise tests. 30 min assessment.
Petersen et al. (2003) [39]	Develop a classification system with pathoanatomic orientation for use in primary care.	Judgemental approach. Physiotherapist authorship. Slightly modified version of Laslett and van Wijmen (1999) [81] classification system.	Non-specific LBP.	Red flag symptoms, hip disorders, suspected referred pain from viscera.	1: Disc syndrome (reducible;irreducable and non-mechanical) 2: Adherent nerve root 3: Nerve root entrapment 4: Nerve root compression 5: Spinal stenosis 6: Zygapophysial joint 7: Postural 8: Sacro-iliac joint 9: Myofascial pain 10: Adverse neural tension 11: Abnormal pain 12: Inconclusive	Patient history and clinical examination.	Some training required and experience of the McKenzie assessment. Takes 1 h to complete.
Vining et al. 2013 [46]	Create a classification system based on available evidence for use in research and clinical setting	Judgement approach. Based on Petersen et al. (2003) [80] model Chiropractic authorship.	LBP.	None	1. Screening 2. Nociceptive - Discogenic - SIJ - Zygapophyseal joint -Myofascial 3. Neuropathic - Compressive radiculopathy - Non compressive radiculopathy - Neurogenic claudication - Central pain 4. Functional instability 5. Other diagnoses	Patient history and clinical examination. Questions and physical component of the Leeds Assessment for Neuropathic Symptoms and Signs (LANSS). Arterial brachial index test for neurogenic claudication if indicated	None.

**Table 5:** Data extraction for classification systems: Systems classifying by Treatment based approach

Primary Author	Purpose	Method of Development	Domain of Interest	Specific Exclusions	Categories	Criteria used	Training/Personnel needed
Delitto et al. (2012) [48]	Classify and define musculoskeletal conditions using the World Health Organisation terminology related to International Classification of Functioning, Disability and Health.	Judgement approach. Content experts appointed by Orthopaedic section of the American Physical Therapy Association.	LBP.	Serious medical conditions.	1: Lumbosacral segmental/somatic dysfunction with mobility deficits 2: Spinal instabilities with movement coordination impairments 3: Flatback syndrome or lumbago due to displacement of disc 4: Of acute low back pain with related (referred) lower extremity pain 5: Lumbago with sciatica 6: Low back pain/strain/lumbago -with related cognitive or affective tendencies 7: Of chronic LBP with related generalized pain Additional axis-Yes-acute, subacute, chronic	Patient history and clinical examination. Questionnaires for category with related cognitive or affective tendencies.	None.
Hall et al. (1994) [55]	Identify typical patterns of pain and determine treatment direction.	Judgement approach. Spinal surgeon and physical therapist authorship.	LBP.	None.	1: LBP +/- referred pain aggravated by flexion, slow onset lasting weeks 2: LBP +/- referred pain aggravated by extension, sudden onset lasts 1–2 weeks 3: Leg dominant pain due to nerve involvement, aggravated by flexion, slow onset, lasts weeks 4: Leg dominant pain due to nerve involvement aggravated by activity and extreme sustained extension, relieved by rest. Rapid onset 5: Abnormal pain behaviour, chronic pattern associated work/sleep/psycho/social issues Additional Axis- No	Patient history and clinical presentation.	None.
McKenzie (1981) [49]	Develop a classification to determine choice of treatment.	Judgement approach. Physiotherapy authorship.	LBP.	Constant pain, serious pathology, neurological deficit.	1: Postural 2: Dysfunction 3: Derangement 1–7	Patient history and clinical examination.	Training in McKenzie assessment desired.
Albert et al. 2012 [61]	Examine the association between treatment outcome and baseline type of disc lesion.	Judgement approach. Physiotherapy authorship.	Radicular pain with dermatomal distribution to knee or below. 176 patients with sciatica involved in large RCT.	>65 years old, leg pain < 3 on 1–10 scale, duration < 2 weeks or > 1 year, red flags, previous back surgery, serious comorbidities.	5 groups based on their pain response: 1: Abolition centralization 2: Reduction centralization 3: Unstable centralization 4: Peripheralization 5: No change	Response to repeated moving testing. Lumbar magnetic resonance imaging (MRI).	Training from McKenzie accredited physiotherapist.

**Table 6:** Data extraction for classification systems: Systems classifying by Screening Tool/Prediction Rule

Primary author	Purpose	Method of development	Domain of interest	Specific exclusions	Categories	Criteria used	Training/Personnel needed
Fritz et al. (2007) [64]	To identify if there is a subgroup of patients likely to respond to traction	Judgement and statistical approach.	LBP with signs of nerve root compression Primary care.	>60 years old, red flags, previous spinal surgery in past 6 months, pregnancy, absence of symptoms when sitting.	Patients likely to benefit from traction have: leg symptoms; signs of nerve root compression; symptom peripheralization on extension movement; positive crossed SLR	Patient history and clinical examination	None.
Roach et al. (1997) [63]	To develop screening tests to place patients into a predetermined structure-based diagnostic classification system.	Judgemental and statistical approach. Physiotherapy authorship.	LBP. 106 tertiary care patients.	Back pain treatment within last year, history of back surgery, unconfirmed diagnosis at end of study.	1: Disk, 2: Spinal stenosis, 3: Disk disease with spinal stenosis 4: Benign low back pain.	Questionnaire (Pain response to activity and position questionnaire). Additional advanced diagnostic tools such as CT/MRI and lab work.	None.
Scholz et al. (2009) [62]	Test the utility of a tool (Standardized Evaluation of Pain (StePs)) to differentiate between radicular and axial pain.	Statistical approach. Anesthesiology and Pharmacology authorship.	Chronic LBP.	Pain < 3 months, <18 years old, global pain intensity in week prior to recruitment <6 severe psychiatric or medical illness, another painful or neurological disease or local infection.	Axial low back pain. Radicular low back pain. Most discriminatory items for radicular pain: positive SLR, deficit in detection of cold and reduced response to pinprick Also identified subtypes of radicular and axial LBP based on clusters of signs and symptoms.	Brief structured interview of 6 questions and 10 standardized physical tests.	Training in administering the tests in physical examination to assess cutaneous changes, pressure; pinprick; vibration; thermal sensitivity and proprioception.

**Table 7:** Data extraction for classification systems: Systems classifying by Pain Mechanisms

Primary author	Purpose	Method of development	Domain of interest	Specific exclusions	Categories	Criteria used	Training/Personnel needed
Schafer et al. 2009 [65]	Identify the predominant pain mechanisms responsible for patients back and leg pain to guide treatment decisions.	Judgement approach. Physiotherapy authorship.	Low back related leg pain.	Recent surgery or nerve root block, diabetes vascular disease in lower extremities, systematic disease. Inflammatory arthropathies.	1. Central sensitization 2. Denervation 3. Peripheral nerve sensitization 4. Musculoskeletal	Patient history and clinical examination. Questions and physical component of the Leeds Assessment for Neuropathic Symptoms and Signs (LANSS).	None.
Smart et al. 2011 [66]	Identify signs and symptoms of patients categorized according to mechanism-based classification of pain.	Judgement and statistical approach. Expert consensus panel to develop clinical criteria list.	LBP +/- leg pain. 464 patients.	History of diabetes, central nervous system injury, pregnancy, non musculo-skeletal LBP.	1. Centralisation pain 2. Peripheral neuropathic 3. Nociceptive	Patient history and clinical examination.	Practical training with an assessment manual provided.
Nijs et al. 2015 [75]	Apply a pain classification system to LBP patients	Judgement approach Expert opinion of 18 international pain experts	LBP	n/a	1. Nociceptive pain 2. Neuropathic pain 3. Central sensitization	Patient history, clinical examination, diagnostic investigations	None

**Table 8:** Overview of classification systems organised by themes and accompanying scores

Clinical features		Pathoanatomy		Treatment based approach		Screening/Prediction tool		Pain mechanisms	
Barker 1990 [79]	2	Bernard and Kirkaldy Willis 1987 [41]	2	Albert et al. 2012 [61]	4	Fritz et al. 2007 [64]	3	Schafer et al. 2009 [65]	5
Ben Debba et al. 2000 [36]	3.5	Cassisi et al. 1993 [38]	3	Hall et al. 1994 [49]	5	Roach et al. 1997 [63]	3	Smart et al. 2011 [66]	5
Glassman et al. 2011 [37]	2.5	Hahne et al. 2011 [38]	3	Mckenzie 1981 [49]	5.5	Scholz et al. 2009 [62]	4	Nijs et al. 2015 [75]	2.5
Nachemson and Andersson 1982 [80]	3.5	Paatelma et al. 2009 [44]	3.5	Delitto et al. 2012 [48]	3.5				
Spitzer et al. 1987 [25]	4	Petersen et al. 2003 [39]	4						
Sweetman et al. 1992 [26]	2.5	Vining et al. 2013 [46]	3.5						

### General summary of classification systems organised by themes

#### Clinical features

Six papers described classification systems according to clinical signs and symptoms (Table 3). They scored low using the appraisal tool (median score 3, interquartile range (IQR) =1). With the exception of the Quebec Task Force Classification (QTFC) system [25], there was no supporting work on the systems’ validity and generalizability. One system [26] used statistical methods to derive clusters of LBP patterns. Development methods for the other five systems were judgement based. Although the QTFC system was judgement based, a multidisciplinary task force representing a wide range of disciplines were engaged to develop the system categories.

The QTFC has been extensively investigated and adapted. The first four categories, which do not involve information from advanced imaging such as magnetic resonance imaging (MRI), have shown good discriminative ability [27] with the more severe/disabling categories associated with poorer function and inability to return to work [28, 29], poorer movement quality [30], higher presence of neuropathic pain [31], less favourable response to treatment [32] and the probability of surgical treatment increasing from category 2 (pain + radiation proximal extremity) to category 6 (spinal nerve root compression confirmed by imaging) [33]. In a Danish study [5], 2673 patients were classified into one of the first four subgroups of the QTFC, and patients with signs of NRI were the ones most severely affected in terms of pain, disability, work participation and psychosocial profile. In a prospective study using the same dataset [34], leg pain, with or without neurological signs, predicted activity limitation and time off work but was not influenced by whether the pain is above or below the knee. One study has compared the QTFC (categories 1 to 4) system to classifying whether leg pain centralized or peripheralised [35]. Both systems could differentiate between groups’ baseline pain intensity and disability, but the classification method by leg pain centralisation or peripheralisation was superior in predicting treatment outcomes and long term work status. Ben Debba et al. [36] used categories 1 to 4 of the 11 category QTFC, with the addition of the straight leg raise test to determine the presence of neurological signs and showed good discriminative ability between the categories.

We did not identify any reliability studies for the QTFC system. Glassman et al. [37] established substantial reliability (kappa = 0.698) among physicians reviewing case histories. Sweetman et al. [26] reported 70 % reproducibility when their classification algorithm was used on a smaller sample of 80 LBP patients.

#### Pathoanatomy

The purpose of the six pathoanatomical classification systems (Table 4) was to identify a pathology or anatomical structure responsible for a person’s LBP. In two of the six systems [38, 39], specific treatments were suggested for the identified categories. Overall, on the appraisal tool, the systems scored low (median score = 3.25, interquartile range (IQR) =0.875) mainly due to lack of supporting work on the systems’ validity and generalisability. All used a judgement approach for development.

The number of categories in all systems ranged from two [40] to twenty-two categories [41] with overlap of identified categories among the six systems. All recognised the lumbar disc as a pain source. Facet joint was included in five of the systems, and four systems included stenosis. Leg pain of radicular origin was considered in all six groups but under varying nomenclature and criteria for diagnosis (Table 9). There was some evidence of supporting validity work. Cassisi et al. [40] explored differences in pain, disability and psychological function between their two groups of myofascial pain and disc herniation. Hahne et al. [38] designed their classification system for use in a planned clinical trial to compare specific physiotherapy treatment to physiotherapy advice for the five subgroups of LBP. Results show a reduction in activity limitation and back and leg pain intensity across a 52-week follow-up for patients who received 10 individual sessions relative to two sessions of guideline-recommended advice [42].

**Table 9:** Terms and clinical criteria used to describe nerve root involvement among the classification systems

Author (first)	Terms to describe nerve root involvement	1	2	3	4	5	6	7	8	9	10	11	12	13	14	15
Clinical features																
Baker 1990 [79]	Sciatica	x					x		x	x	x	x				
Ben Debba et al. 2000 [36]	Back and below knee pain with positive SLR	x										x				
Glassman et al. 2011 [37]	Leg pain dominant; neurogenic claudication					x		x								
Nachemson 1982 [80]	Sciatica; Rhizopathy		x	x	x				x	x	x	x				
Spitzer et al. 1987 [25]	Pain with lower limb radiation with neurological signs; Spinal stenosis	x							x	x	x	x				
Sweetman et al. 1992 [26]	Sciatica	x	x							x	x	x				
Pathoanatomy																
Bernard 1987 [41]	Herniated nucleus pulposis; Spinal stenosis	x	x	x		x	x		x	x	x	x	x			
Cassisi et al. 1993 [40]	Disc herniation	x	x			x			x	x	x	x				
Hahne et al. 2011 [38]	Disc herniation with radiculopathy	x							x	x	x	x				x
Paatelma et al. 2009 [44]	Discogenic pain with nerve root irritation; Spinal stenosis							x				x				
Petersen et al. 2003 [39]	Disc syndrome:reducible/irreducible							x	x	x	x	x		x		
Vining et al. 2013 [46]	Radiculopathy: non/compressive; Neurogenic claudication			x		x		x		x	x			x	x	
Treatment approach																
Delitto et al. 2012 [48]	Lumbago with sciatica				x				x	x	x	x		x		
Hall et al. 1994 [55]	Leg dominant pain due to nerve root involvement					x		x	x	x	x	x		x		
McKenzie 1981 [49]	Derangement; Adherent nerve root				x									x		
Albert et al. 2012 [61]	Sciatica	x	x						x	x	x	x				
Screening tools/CPR																
Fritz et al. 2007 [64]	Low back pain with signs of nerve root involvement								x	x	x	x				
Roach et al. 1997 [63]	Disc; Spinal stenosis															x
Scholz et al. 2009 [62]	Radicular pain									x		x			x	
Pain mechanisms																
Smart et al. 2011 [66]	Peripheral neuropathic	x	x									x				
Schafer et al. 2009 [65]	Denervation; Peripheral nerve sensitization		x		x				x	x	x	x			x	
Nijs et al. 2015 [75]	Neuropathic/radicular pain	x	x		x		x		x						x	x
Key for history and clinical examination criteria for sciatica																
1 Pain below knee 2 Dermatomal distribution of symptoms 3 Positive cough/sneeze 4 Pins & needles/numbness: subjective reporting 5 Leg pain worse than back pain	6 Quality descriptor of pain eg “burning” 7 Stenotic aggravating/easing factors 8 Sensory deficit in lower limb (LL) objectively 9 Strength deficit in LL objectively 10 Altered LL reflexes	11 Positive neural tension tests 12 Positive crossed straight leg raise 13 Aggravated with specific lumbar range of movement 14 Other 15 Positive findings from imaging eg MRI														

Petersen et al. [39] detailed content validity for several of their categories. A follow-up study showed that six of the most common pathoanatomical categories in their system, diagnosed by physiotherapists in chronic LBP patients, had low agreement (kappa of 0.31) with chosen reference standards (advanced imaging tests, injections or discography) [43]. Two systems were tested for reliability among clinicians and reported good inter-rater reliability [44, 45]. Petersen et al. [39] acknowledged that their system was difficult to perform and would take up to one hour. Paatelma et al. [44] reported their system took 30 min to subdivide patients into one of its 5 categories. These were the only 2 out of all 22 classification systems that gave information on how long the assessments would take to perform. Both these systems and the one by Hahne et al. [38] required training for the clinicians using them.

A group of Canadian chiropractors created a diagnostic classification system based on available evidence [46]. They began with Petersen et al’s [39] pathoanatomical system and modified some of the categories, but also added or updated diagnostic criteria based on available evidence. No validation or reliability work has been subsequently published to support their system, but they do base many of their diagnostic criteria on available statistically derived diagnostic models. For example, compressive radiculopathy is a subcategory within a neuropathic pain category. They used clinical assessment items identified from a statistically derived diagnostic tool [47] and added the score from a neuropathic pain tool (Leeds Assessment of Neuropathic Symptoms and Signs (LANSS)) score to assign patients to this category.

#### Treatment-based approach

The four treatment-based classification systems (Table 5) are designed to guide specific treatment allocation to LBP patients. They scored a median of 4.5 (IQR 1.5) points. All systems were developed based on the judgement approach of the authors. Delitto et al’s classification [48] involved an expert panel.

In the McKenzie system [49], also known as Mechanical Diagnosis and Therapy (MDT), patients are classified according to how their symptoms respond to repeated spinal movements and sustained positions [50]. Since its development in 1981 several studies have been published supporting its validity, reliability and generalisability. A systematic review and meta-analysis [51] evaluating the effectiveness of the McKenzie approach concluded that in patients with acute LBP, the McKenzie method produces similar improvements in pain or disability as passive therapy and advice to stay active. In a later RCT where all patients received information, advice and either manipulation or McKenzie based treatment, a more favourable outcome was seen with the McKenzie approach at 2 months follow up [52]. Subgroup analysis based on this RCT showed that peripheralisation of leg symptoms (towards the feet) and NRI were predictors of good treatment outcome with the Mckenzie treatment. The authors reflect that peripheralisation of symptoms was not the expected direction and acknowledge patient numbers were small and confidence intervals wide [53]. A review concluded that there is high strength of evidence of substantial agreement among clinicians certified (formally trained and successfully completing an examination) in the McKenzie approach for classifying patients [24]. A more recent reliability study involving 1662 patients and 47 raters indicated that inter-rater reliability was not acceptable for therapists at any level of McKenzie training [54].

Hall et al. [55] described five patterns of LBP including leg pain, with the dominant pattern determining the appropriate treatment. Despite basing their classification on clinical assessment, the authors offer explanations for patients of ‘painful disc’, ‘worn spinal joints’, ‘pinched nerve’ and ‘bony spurs within the spine’ for categories I to IV respectively. A later study [56] compared outcomes in patients classified according to their system, with patients managed without a classification system and concluded that classification had a positive effect on pain relief post treatment, resulted in less treatment days and patients were less likely to use pain medication. Weaknesses of this validation study included use of a double - cohort study design, i.e. comparison of two cohorts and not a randomised controlled trial, which meant significant differences at baseline between the usual care groups and the classified group. The intervention for the non-classified patients was also poorly described. The reliability of the Hall et al’s [55] system was good (kappa =0.6) [57]. It is currently being implemented in the Canadian province of Saskatchewan as part of the Saskatchewan clinical spine pathway (SSP) to manage patients with LBP in primary care [58]. Based on a sample of 87 LBP patients, implementation of the pathway showed reduction in MRI utilisation and referrals seen by surgeons for nonoperative care, suggesting a potential for cost savings [59] and the process suggests reduction in waiting times and costs by timely direction of suitable patients for surgical review [60]. Further studies are underway to assess the efficacy of the SSP.

Albert et al. [61] classified patients with sciatica (radicular pain) according to whether their leg pain centralized, peripheralised or did not change. Other sources refer to this classification as pain pattern classification [35]. All patients received exercise and advice. Similar improvements in activity limitation and leg pain were seen in those who were categorised as ‘centralisers’ and in those whose symptoms ‘peripheralised’, which refutes some of the proposed McKenzie model theory. Additionally, symptoms centralized in over 90 % of patients with MRI confirmed sequestrated or extruded discs.

Clinical guidelines published by the Orthopaedic group of the American Physical Therapy Association [48] proposed a function/impairment based classification for LBP. The system classifies patients with and without leg pain into eleven mutually exclusive impairment patterns. Each category has a recommended treatment approach. Although no supporting work was identified in the review that examines reliability, validity or generalizability of the system, the authors designed the treatment system based on the validation work done by others.

#### Screening tools/Clinical prediction rules

Three papers were grouped under screening tools and prediction rules (Table 6), scoring a median of 3 points (IQR 1). All combined judgement and statistical approaches to system development but had limited follow-up supporting studies. The purpose was to identify clinical features that either guide diagnosis [62, 63], or assist with treatment selection [64]. They are grouped together due to similar concepts and methodology. Scholz et al. [62] used statistical analysis to identify the most discriminatory items from a neuropathic pain assessment tool (Standardized Evaluation of Pain (StEP)) to differentiate between LBP patients with and without radicular leg pain. Using cluster analysis they also identified 4 subtypes with similar pain patterns. Roach et al. [63] developed screening test algorithms, based on patients’ answers to a Pain Response to Activity and Position questionnaire, to place patients into four predetermined “structure-based” diagnostic classifications. A judgement approach method was used to preselect the four LBP categories of disc; spinal stenosis; disc disease with spinal stenosis and benign LBP. Fritz et al. [64] identified a subgroup of LBLP patients with signs of nerve root compression, likely to respond to mechanical traction and found that baseline variables associated with greater improvements with traction were peripheralisation of leg symptoms with extension movement and a cross-over SLR. Subsequent validation of the algorithms in Roach et al’s study [63] led to misclassification of a substantial number of patients. Scholz et al’s [62] tool identified patients with radicular pain with high sensitivity and specificity. No published work was available on application of the rules to different population groups. The reliability of the reference standard or diagnostic categories was not tested in any of the studies. Test retest reliability of Roach et al’s [63] screening algorithms had kappa values ranging from 0.57 to 0.91, for the 4 diagnostic categories, suggesting good to almost perfect reliability.

#### Pain mechanisms

Three pain mechanism classification system studies were identified (Table 7) scoring a median 5 points (IQR 2.5). Schafer et al. [65] specifically designed their system for LBLP patients. All systems were initially developed using a judgement approach. Smart et al. [66] subsequently used statistical analysis to identify discriminatory clusters of signs and symptoms associated with each of the categories. There is considerable overlap in the categories proposed by the three systems.

Smart et al ‘s [66] system has three categories: (i) Central sensitisation pain (CSP) (ii) Peripheral neuropathic pain (PNP) and (iii) Nociceptive pain (NP). Standardised clinical interview and examination are used to categorise patients plus a number of additional pain response symptoms (e.g. spontaneous paroxysmal pain and dysesthesia) and physical signs such as allodynic response and painful response to nerve palpation [67]. Supporting the discriminant validity of their system, the authors showed that the CSP group had the most self-report pain, disability, anxiety and depression and poorest health related quality of life compared to the PNP and NP group [68]. A similar pattern was seen between the PNP and NP groups with the PNP group having poorer outcomes compared to the NP group. Schafer et al. [65] described a four category system: (i) Central sensitisation (renamed in a later paper as “Neuropathic sensitisation (NS)” [69]); (ii) Denervation; (iii) Peripheral nerve sensitisation (PNS), and (iv) Musculoskeletal. The LANSS neuropathic self-report pain scale score was used for all four categories. 77 LBLP patients were classified according to the Schafer et al system and all had 7 sessions of neural mobilisation [69]. As hypothesised, improvement in outcomes was greatest for the PNS group supporting the predictive validity of one of their classification subgroups. Another study showed that the PNS group had greater disability than all groups and more fear avoidance beliefs compared to central sensitisation and denervation groups [70]. This was considered a surprising outcome as these results would have been expected more from the central sensitisation group and suggestive that the criteria for the described classification schemes do not clearly differentiate between the three subgroups [71]. Further work to demonstrate the construct validity of their system showed differences in pain hypersensitivity as measured by Quantitative Sensory Testing (QST) between the Neuropathic pain and Denervation groups compared to controls [72]. However, no significant differences were found between the four pain groups which the authors recognised as weakening the construct validity of the classification system. Inter-rater reliability was reported for both of these systems. In 40 patients with LBLP, reliability was substantial (kappa = 0.72, 95 % CI 0.57–0.86) among five pairs of examiners using the Schafer et al classification [73]. Smart et al. [74] used two examiners and reliability was substantial (kappa = 0.77, 95 % CI 0.56–0.96). Intra-rater reliability was almost perfect (kappa = 0.96, 95 % CI = 0.92–1.0) with the developer of the system re-examining the patients within 6–56 days of their initial assessment.

The three pain mechanism groups of nociceptive, neuropathic and central sensitisation are the basis of the third pain mechanism system [75] and, based on a consensus approach of pain experts, the authors apply the criteria for each category to back pain. The authors state that chronic lumbar radicular pain is the most common neuropathic pain syndrome and apply classification criteria for neuropathic pain to LBP. These screening criteria include sensory testing (response to vibration/temperature, pin prick), evidence from diagnostic investigations such as MRI and pain extending below the knee. It is difficult to interpret where patients fit in the classification system if they have some of the neuropathic symptoms i.e. below knee pain, dermatomal pain distribution, burning/shooting/prickling pain but do not score positively on the QST tests. The paper focused predominantly on identification and treatment options for the central sensitisation pain group using criteria from Smart et al’s [66] work.

### Summary of classification systems

**Fig. 2:**
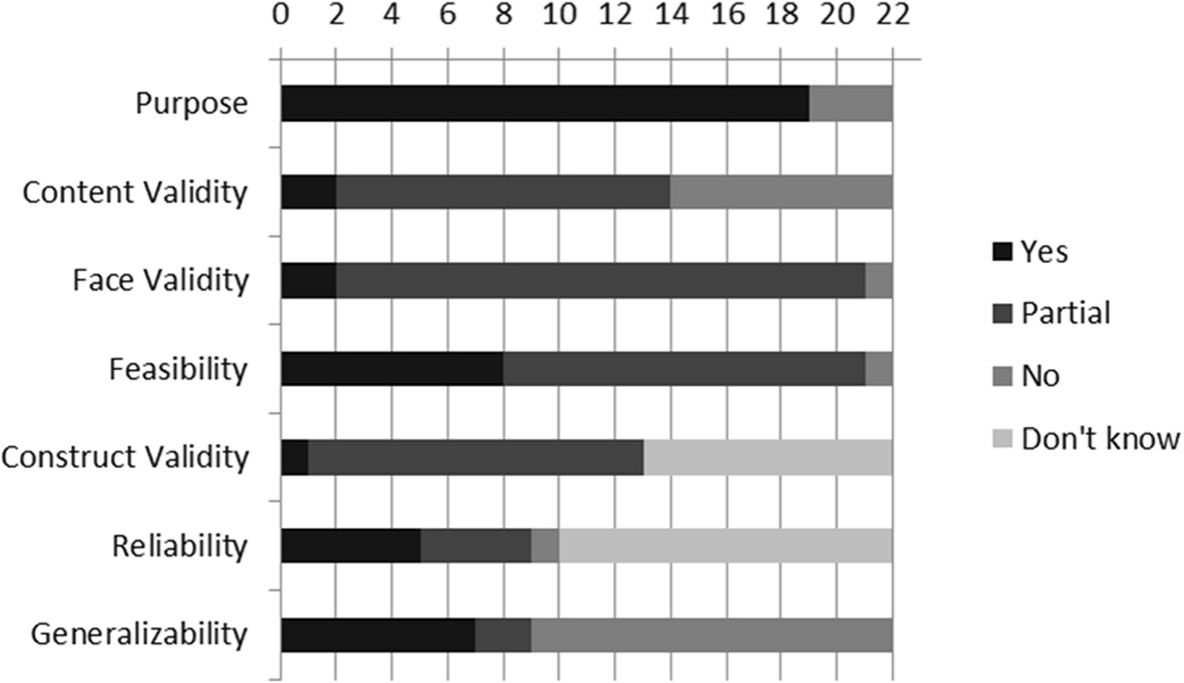
Methodological quality summary of the 22 classification systems based on the appraisal tool

A summary of the strengths and weaknesses of the 22 classification systems based on the quality appraisal scoring tool is presented in Fig. 2. The majority of the systems were clear on the purpose of their classification system. Validity of the systems scored poorly, in particular content and construct validity. Reliability data was available on a small number of the systems and only two commented on the feasibility of their approach. Judging the generalisability of the systems was limited. There was evidence of some of the systems being used in different settings but mainly to test issues of validity and reliability. Only one system [55] is currently being implemented in primary care.

### How is LBLP due to NRI described and diagnosed?

The terminology used for categories with NRI within the classification systems was listed to assess consistency of terms and the clinical criteria within these categories were explored. This is presented in Table 9. Up to 11 different terms were used to describe NRI presentations. The most frequently used terms were sciatica, nerve root, disc and spinal stenosis. But within these terms there was variation, for example nerve root was described as involvement, adherent, compression or irritation. Table 9 quantifies how often features from history and physical examination were used in total by the 22 classification systems. Ten of the 22 systems mentioned “pain below the knee”; 5 out of 22 used “patient’s leg pain was greater than the back pain”. Findings from clinical examination also showed considerable variability. Neurological deficits and positive neural tension tests were both mentioned in 14 of the 22 systems, but criteria varied from being quite prescriptive, specifying at least one of reflex, sensory or muscle strength deficit, to being quite vague with phrases such as “may have” neurological deficits.

## Discussion

Following a comprehensive systematic search of the literature, 22 systems were identified that classified patients with back and leg pain. Only three of the systems focused specifically on LBLP patients [61, 64, 65]. There was a lack of consistency between classification systems when describing NRI and its clinical attributes. The definitions and diagnostic criteria for NRI varied widely among the systems, which mirrors findings from recent reviews on eligibility criteria in studies involving LBLP patients [16, 17]. Consensus on how to define leg pain due to NRI and agreement on clinical criteria to distinguish patients with spinal NRI is needed. If eligibility criteria were more consistent across studies this would enhance communication with patients and among clinicians when discussing diagnosis and possible treatment outcomes (16–18).

The call to identify clinically relevant subgroups of LBP patients has been a top priority in primary care back pain research since the mid-1990s, and researchers have responded accordingly with a proliferation of subgroup studies [2]. Several published reviews have appraised LBP classification systems, each with a slightly different focus, but primarily the emphasis has been on non-specific low back pain classification. This is the first review to look specifically at the classification of patients with LBLP.

The quality of the 22 systems varied, and those that scored higher on the appraisal tool were ones with evidence of more robust methods of development and more supporting published work on reliability, validity and generalisability. The majority of the systems used a judgement approach to development, ranging from authors opinion to expert consensus panels. Some systems used statistical methods to identify clusters of symptoms that best discriminate between patients, giving objective means of identifying subgroups of patients which helps to avoid author bias. Relying on statistical clustering in isolation can give rise to content validity issues, with subgroups not clinically recognisable or identifiable. Using a combined approach of judgement, preferably with group consensus and statistical methods to identify subgroups, is recommended [76] but only one system did this [66]. Among the systems of classifying according to clinical features, the QTFC system [25] scored highest on the quality appraisal tool. It has been extensively investigated, validated and adapted and has widespread application in research with many studies using the first four categories to explore differences among groups and investigate their prognosis. For these reasons, and considering its simplicity and brevity, it seems well placed for use in primary care. However to enhance consistency of this classification, it does require more detailed clarification of the clinical criteria for neurological involvement in category 4.

Pathoanatomical classification systems generally scored low on the appraisal tool, primarily because development was mainly based on authors’ opinion. Many consider the pathoanatomical approach, which seeks to associate specific structures with symptoms, as outdated and unhelpful to patients. It is thought to lead to overuse of diagnostic procedures with subsequent implications on cost and patients’ expectations if findings do not match clinical symptoms [58]. A review of recommendations for LBP clinical practice [77] noted that none of the guidelines recommend that clinicians should attempt to identify specific anatomical structures involved in LBP once potentially serious spinal pathology, specific causes and substantial neurological involvement have been ruled out. Others argue that identification of a cause for LBP is important for patients and the main reason for seeing a primary care practitioner [40]. Neglecting patients’ expectations can impact negatively on patient satisfaction and ‘diagnostic uncertainty’, or inadequate explanation of cause, can lead to higher levels of depression [78] and fear avoidance beliefs [3] in LBP patients.

The treatment based approach classification systems included the McKenzie system which is a popular treatment based approach among clinicians, despite evidence that it is not superior to other treatments. An additional three papers were grouped under screening tools and prediction rules, where statistical methods were used to identify cluster of items to assist diagnosis or prognosis. Classification according to pain mechanisms is gaining popularity in musculoskeletal medicine and three papers applied this system to LBLP [65, 66, 75]. Shafer and colleagues [65] designed their system specifically for LBLP patients. Some confusion arises comparing the nomenclature and criteria of the pain mechanism subgroups. Leg pain with NRI was categorised as denervation or peripheral sensitisation by Schafer et al. [65], as peripheral neuropathic pain by Smart et al. [66] and predominantly neuropathic pain by Nijs et al. [75]. All had different clinical criteria. Schafer’s system [65] has made good efforts to validate their system but has struggled to demonstrate discriminative validity of the categories. This may reflect the judgement based development process of the system. A more robust method including statistical techniques and consensus could serve to improve the validity of the system and assign criteria that allow clearer differentiation between the subgroups.

Smart et al. [67] used statistical methods to identify three items from history and physical examination items that were predictive of peripheral neuropathic: history of nerve injury, pathology or compromise; pain in a dermatomal distribution and positive neurodynamic tests. They recognized that these items differ considerably from criteria found in neuropathic pain screening tools and reflect that it may be because their patients were recruited from primary care settings with less severe presentations than the more severe pain populations in studies from which these questionnaires were derived.

Schafer et al. [65] defined their denervation group as patients with at least two neurological deficits (motor, sensory or reflex). Yet despite these neurological deficits indicative of nerve root compromise, this category also includes a LANSS screening tool score of less than 12, indicative of a low probability of neuropathic pain, at least by self-report. Contrary to this, the categories of compressive and non-compressive radiculopathy in Vining et al’s [46] pathoanatomical system, have a LANSS score of 12 or over, suggesting all radicular pain has a high probability of being neuropathic. Nijs et al. [75] have different screening criteria for neuropathic pain in LBP which includes confirmation of a nervous system abnormality with diagnostic testing e.g. EMG or imaging.

### Strengths and limitations

This is the first review that has focused specifically on classification of LBLP. The search identified over 13,000 citations for initial screening. This large number reflects the breadth of the search strategy and large number of databases searched with minimal restrictions. The broad search strategy was deemed necessary to include all possible terms that could be used to describe LBLP and classification and avoid missing any systems. The search strategy was supplemented by first author searches and hand searching reference lists. Identified systems were not excluded on the basis of quality and study appraisal was systematically and independently carried out by two reviewers. Other systems or supporting evidence may have been missed e.g. unpublished student studies or cases of publication bias if findings were unsupportive of the system.

References were initially missed and 21 of the 50 papers in the systematic review were identified through supplementary search strategies. Despite the comprehensive search strategy which included up to 34 terms to describe LBLP, a possible reason for missing several papers is because of the vast nomenclature used to describe and identify LBLP.

## Conclusion

A primary aim of identifying groups of patients with similar characteristics or diagnostic entities is to guide management, as homogeneous groups may respond more favourably to certain management options. However, the first step is to be able to identify these groups with reasonable diagnostic certainty based on generally accepted characteristics.

The classification of LBLP merits more attention, especially in primary care settings where most of these patients are assessed and managed. This should start with agreement on the criteria that reasonably distinguishes NRI from pain referred into the leg from structures in the back other than the nerve root. An approach that uses data from large, unselected groups of primary care patients to classify them according to relevant characteristics from self-reported measures, clinical examination findings and perhaps even demographic information, deserves more attention to appreciate the clinical characteristics of this subgroup of LBP patients. This methodology has been used more often in systems to subgroup psychosocial characteristics in chronic LBP patients [22] and it is equally applicable in LBLP classification.

A greater understanding of the profile of LBLP patients could help shape future research questions and directions in this subgroup of patients in terms of prognostic and effectiveness studies.

### Additional editorial policies

Research involving animals: not applicable.

Research involving plants: not applicable.

Trial registration: not applicable. The systematic review protocol was not registered.

Standards of reporting: The PRISMA guidelines for reporting systematic reviews were followed.

Describing new taxa: not applicable.

## Additional files

Additional file 1: Medline Search Strategy. (DOCX 17 kb) Additional file 2: Summary of the critical appraisal for all papers. (DOCX 23 kb)

## Abbreviations

LANSS: Leeds assessment of neuropathic symptoms and signs
LBLP: Low back-related leg pain
MRI: Magnetic resonance imaging
NRI: Nerve root involvement
QTFC: Quebec task force classification
RCT: Randomised controlled trial

## References

[1] Hoy D, March L, Brooks P, Blyth F, Woolf A, Bain C, et al. The global burden of low back pain: estimates from the Global Burden of Disease 2010 study. Ann Rheum Dis. 2014;73(6):968–74. 10.1136/annrheumdis-2013-204428 24665116

[2] Costa Lda C, Koes BW, Pransky G, Borkan J, Maher CG, Smeets RJ (2013). Primary care research priorities in low back pain: an update. Spine.

[3] Waddell G (2004). The back pain revolution.

[4] Hill JC, Konstantinou K, Egbewale BE, Dunn KM, Lewis M, van der Windt D (2011). Clinical outcomes among low back pain consulters with referred leg pain in primary care. Spine.

[5] Kongsted A, Kent P, Albert H, Jensen TS, Manniche C (2012). Patients with low back pain differ from those who also have leg pain or signs of nerve root involvement - a cross-sectional study. BMC Musculoskel Disord.

[6] Merskey H, Bogduk N (1994). Classification of chronic pain.

[7] Fransen M, Woodward M, Norton R, Coggan C, Dawe M, Sheridan N (2002). Risk factors associated with the transition from acute to chronic occupational back pain. Spine.

[8] Shaw WS, Pransky G, Fitzgerald TE (2001). Early prognosis for low back disability: intervention strategies for health care providers. Disabil Rehabil.

[9] Konstantinou K, Hider SL, Jordan JL, Lewis M, Dunn KM, Hay EM (2013). The Impact of Low Back-related Leg Pain on Outcomes as Compared With Low Back Pain Alone: A Systematic Review of the Literature. Clin J Pain.

[10] Haswell K (2008). Clinical Decision Rules for Identification of Low Back Pain Patients With Neurologic Involvement in Primary Care. Spine.

[11] Valat J, Genevay S, Marty M, Rozenberg S, Koes B (2010). Sciatica. Best Practice & Research Clinical Rheumatology.

[12] Bogduk N, McGuirk B (2002). Medical management of acute and chronic low back pain : an evidence-based approach.

[13] Bogduk N (2009). On the definitions and physiology of back pain, referred pain, and radicular pain. Pain.

[14] Vroomen PC, de Krom MC, Knottnerus JA (2000). Consistency of history taking and physical examination in patients with suspected lumbar nerve root involvement. Spine.

[15] Fairbank J (2007). Sciatica: an archaic term. BMJ.

[16] Lin C, Verwoerd A, Maher C, Verhagen A, Pinto R, Luijsterburg P, et al. How is radiating leg pain defined in randomized controlled trials of conservative treatments in primary care? A systematic review. Eur J Pain. 2014;18:455–64. 10.1002/j.1532-2149.2013.00384.x 23939653

[17] Genevay S, Atlas SJ, Katz JN (2010). Variation in eligibility criteria from studies of radiculopathy due to a herniated disc and of neurogenic claudication due to lumbar spinal stenosis: A structured literature review. Spine.

[18] Konstantinou K, Dunn KM (2008). Sciatica: review of epidemiological studies and prevalence estimates. Spine.

[19] Buchbinder R, Goel V, Bombardier C, Hogg-Johnson S (1996). Classification systems of soft tissue disorders of the neck and upper limb: Do they satisfy methodological guidelines?. J Clin Epidemiol.

[20] Riddle DL (1998). Classification and low back pain: a review of the literature and critical analysis of selected systems. Phys Ther.

[21] Petersen T, Thorsen H, Manniche C, Ekdahl C (1999). Classification of non-specific low back pain: a review of the literature on classifications systems relevant to physiotherapy. Phys Ther Rev.

[22] McCarthy CJ, Arnall FA, Strimpakos N, Freemont A, Oldham JA (2004). The biopsychosocial classification of non-specific low back pain: a systematic review. Phys Ther Rev.

[23] Billis EV, McCarthy CJ, Oldham J (2007). Subclassification of low back pain: a cross country comparison. Eur Spine J..

[24] Fairbank J, Gwilym S, France J, Daffner S, Dettori J, Hermsmeyer J, et al. The role of classification of chronic low back pain. Spine. 2011;36:S19–42. 10.1097/BRS.0b013e31822ef72c 21952188

[25] Spitzer WO, LeBlanc FE, Dupuis M (1987). Scientific approach to the assessment and management of activity related spinal disorders. A monograph for clinicians. Report of the Quebec Task Force on Spinal Disorders. Spine.

[26] Sweetman BJ, Heinrich I, Anderson JAD (1992). Clinical tests and patterns of low back pain. Journal of Orthop Rheum.

[27] Frank AO, De LH, McAuley JH, Sharma V, Main CJ (2000). A cross-sectional survey of the clinical and psychological features of low back pain and consequent work handicap: use of the Quebec Task Force classification. Int J Clin Pract.

[28] Loisel P, Vachon B, Lemaire J, Durand MJ, Poitras S, Stock S, et al. Discriminative and predictive validity assessment of the quebec task force classification. Spine. 2002;27(8):851–7. 10.1097/00007632-200204150-00013 11935108

[29] Hearne MA (1997). Physical therapy treatment of low back pain: A report of outcomes according to three types of patients classification. J Rehab Outcomes measures..

[30] Marras WS, Parnianpour M, Ferguson SA, Kim JY, Crowell RR, Bose S (1995). The classification of anatomic and symptom based low back disorders using motion measure models. Spine..

[31] Attal N, Perrot S, Fermanian J, Bouhassira D (2011). The neuropathic components of chronic low back pain: a prospective multicenter study using the DN4 Questionnaire. J Pain.

[32] DeRosa CP, Porterfield JA (1992). A physical therapy model for the treatment of low back pain. Phys Ther.

[33] Atlas SJ, Deyo RA, Patrick DL, Convery K, Keller RB, Singer DE (1996). The Quebec Task Force classification for Spinal Disorders and the severity, treatment, and outcomes of sciatica and lumbar spinal stenosis. Spine.

[34] Kongsted A, Kent P, Jensen TS, Albert H, Manniche C (2013). Prognostic implications of the Quebec Task Force classification of back-related leg pain: an analysis of longitudinal routine clinical data. BMC Musculoskel Disord.

[35] Werneke MW, Hart DL (2004). Categorizing patients with occupational low back pain by use of the Quebec Task Force Classification system versus pain pattern classification procedures: discriminant and predictive validity. Phys Ther.

[36] BenDebba M, Torgerson WS, Long DM (2000). A validated, practical classification procedure for many persistent low back pain patients. Pain.

[37] Glassman SD, Carreon LY, Anderson PA, Resnick DK (2011). A diagnostic classification for lumbar spine registry development. Spine J.

[38] Hahne AJ, Ford JJ, Surkitt LD, Richards MC, Chan AY, Thompson SL, et al. Specific treatment of problems of the spine (STOPS): design of a randomised controlled trial comparing specific physiotherapy versus advice for people with subacute low back disorders. BMC Musculoskel Disord. 2011;12:104. 10.1186/1471-2474-12-104 PMC3121656 21599941

[39] Petersen T, Laslett M, Thorsen H, Manniche C, Ekdahl C, Jacobsen S (2003). Diagnostic classification of non-specific low back pain. A new system integrating patho-anatomic and clinical categories. Physiotherapy Theory and Practice.

[40] Cassisi JE, Sypert GW, Lagana L, Friedman EM, Robinson ME (1993). Pain, disability, and psychological functioning in chronic low back pain subgroups: myofascial versus herniated disc syndrome. Neurosurgery.

[41] Bernard JTN, Kirkaldy-Willis WH (1987). Recognizing specific characteristics of nonspecific low back pain. Clin Orthop Relat Res.

[42] Ford J, Hahne A, Surkitt L, Chan AY, Richards M, Slater S, Hinman R, Pizzari T, Davidson M, Taylor N. Individualised physiotherapy as an adjunct to guideline based advice for low back disorders in primary care: a randomised controlled trial. Br J Sports Med. 2015. doi:10.1136/bjsports-2015-095058. 10.1136/bjsports-2015-095058 26486585

[43] Laslett M, McDonald B, Tropp H, Aprill CN, Oberg B. Agreement between diagnoses reached by clinical examination and available reference standards: a prospective study of 216 patients with lumbopelvic pain. BMC Musculoskelet Disord. 2005;6:28–8. 10.1186/1471-2474-6-28 PMC1184083 15943873

[44] Paatelma M, Karvonen E, Heinonen A (2009). Inter-tester reliability in classifying acute and subacute low back pain patients into clinical subgroups: a comparison of specialists and non-specialists. A pilot study. J Man Manip Ther.

[45] Petersen T, Olsen S, Laslett M, Thorsen H, Manniche C, Ekdahl C, et al. Inter-tester reliability of a new diagnostic classification system for patients with non-specific low back pain. Aust J Physiother. 2004;50(2):85–94. 10.1016/s0004-9514(14)60100-8 15151492

[46] Vining R, Potocki E, Seidman M, Morgenthal AP (2013). An evidence based diagnostic classification system for low back pain. J Can Chiropr Assoc..

[47] Vroomen PC, de Krom MC, Wilmink JT, Kester AD, Knottnerus JA (2002). Diagnostic value of history and physical examination in patients suspected of lumbosacral nerve root compression. J Neurol, Neurosur Psychiatry..

[48] Delitto A, George SZ, Van Dillen LR, Whitman JM, Sowa G, Shekelle P (2012). Low back pain clinical practice guidelines linked to the International Classification of Functioning, Disability, and Health from the Orthopaedic Section of the American Physical Therapy Association [with consumer summary]. J Orthop Sports Phys Ther.

[49] Mckenzie RA (1981). The Lumbar Spine: Mechanical Diagnosis and Therapy : Waikanae.

[50] Hefford C (2008). McKenzie classification of mechanical spinal pain: Profile of syndromes and directions of preference. Man Ther.

[51] Machado LAC, de Souza MS, Ferreira PH, Ferreira ML (2006). The McKenzie method for low back pain: a systematic review of the literature with a meta-analysis approach [with consumer summary]. Spine.

[52] Petersen T, Larsen K, Nordsteen J, Olsen S, Fournier G, Jacobsen S (2011). The McKenzie Method Compared With Manipulation When Used Adjunctive to Information and Advice in Low Back Pain Patients Presenting With Centralization or Peripheralization: A Randomized Controlled Trial. Spine.

[53] Petersen T, Christensen R, Juhl C (2015). Predicting a clinically important outcome in patients with low back pain following McKenzie therapy or spinal manipulation: a stratified analysis in a randomized controlled trial. BMC Musculoskelet Disord..

[54] Werneke M, Deutscher D, Hart D, Stratford P, Ladin J, Weinberg J, et al. McKenzie Lumbar Classification: Inter-rater Agreement by Physical Therapists With Different Levels of Formal McKenzie Postgraduate Training. Spine. 2014;39(3):E182–90. 10.1097/BRS.0000000000000117 24253786

[55] Hall H, McIntosh G, Melles T (1994). A different approach to back pain diagnosis: identifying a pattern of pain. Canadian journal of CME..

[56] Hall H, McIntosh G, Boyle C (2009). Effectiveness of a low back pain classification system. Spine J..

[57] Wilson L, Hall H, McIntosh G, Melles T (1999). Intertester reliability of a low back pain classification system. Spine.

[58] Fourney DR, Dettori JR, Hall H, Hartl R, McGirt MJ, Daubs MD (2011). A systematic review of clinical pathways for lower back pain and introduction of the Saskatchewan spine pathway [with consumer summary]. Spine.

[59] Kindrachuk DR, Fourney DR (2014). Spine surgery referrals redirected through a multidisciplinary care pathway: effects of nonsurgeon triage including MRI utilization. J Neurosurg Spine.

[60] Wilgenbusch CS, Wu AS, Fourney DR (2014). Triage of spine surgery referrals through a multidisciplinary care pathway: a value based comparison with conventional referral processes. Spine..

[61] Albert HB, Hauge E, Manniche C (2012). Centralization in patients with sciatica: are pain responses to repeated movement and positioning associated with outcome or types of disc lesions?. Eur Spine J.

[62] Scholz J, Mannion RJ, Hord DE, Griffin RS, Rawal B, Zheng H, et al. A Novel Tool for the Assessment of Pain: Validation in Low Back Pain. PLoS Med. 2009;6(4), e1000047. 10.1371/journal.pmed.1000047 PMC2661253 19360087

[63] Roach KE, Brown MD, Albin RD, Delaney KG, Lipprandi HM, Rangelli D (1997). The sensitivity and specificity of pain response to activity and position in categorizing patients with low back pain. Phys Ther.

[64] Fritz JM, Lindsay W, Matheson JW, Brennan GP, Hunter SJ, Moffit SD, et al. Is there a subgroup of patients with low back pain likely to benefit from mechanical traction? Results of a randomized clinical trial and subgrouping analysis. Spine. 2007;32(26):E793–800. 10.1097/BRS.0b013e31815d001a 18091473

[65] Schäfer A, Hall T, Briffa K (2009). Classification of low back-related leg pain-A proposed patho-mechanism-based approach. Man Ther.

[66] Smart KM, Blake C, Staines A, Doody C (2011). The Discriminative Validity of "Nociceptive," " Peripheral Neuropathic," and "Central Sensitization" as Mechanisms-based Classifications of Musculoskeletal Pain. Clin J Pain.

[67] Smart KM, Blake C, Staines A, Thacker M, Doody C (2012). Mechanisms-based classifications of musculoskeletal pain: Part 2 of 3: Symptoms and signs of peripheral neuropathic pain in patients with low back (±leg) pain. Man Ther.

[68] Smart KM, Blake C, Staines A, Doody C (2012). Self-reported pain severity, quality of life, disability, anxiety and depression in patients classified with 'nociceptive', 'peripheral neuropathic' and 'central sensitisation' pain. The discriminant validity of mechanisms-based classifications of low back (+/- leg) pain. Man Ther.

[69] Schäfer A, Hall T, Mueller G, Briffa K (2011). Outcomes differ between subgroups of patients with low back and leg pain following neural manual therapy: a prospective cohort study. Eur Spine J.

[70] Walsh J, Hall T (2009). Classification of low back-related leg pain: do subgroups differ in disability and psychosocial factors?. J Man Manip Ther.

[71] O’Hearn M, Lowry C, Emerson-Kavchak A, Courtney C (2009). Letter to the editor Re: “Interrater reliability of a new classification system for patients with neural low back-related leg pain”. Journal of Manual & Manipulative Therapy 17(2), 109-118. J Man Manip Ther.

[72] Schäfer A, Hall TM, Rolke R, Treede RD, Lüdtke K, Mallwitz J, et al. Low back related leg pain: an investigation of construct validity of a new classification system. J Back Musculoskelet Rehabil. 2014;27:409–18. 10.3233/BMR-140461 24614828

[73] Schäfer A, Hall TM, Lüdtke K, Mallwitz J, Briffa NK (2009). Interrater reliability of a new classification system for patients with neural low back-related leg pain. J Man Manip Ther.

[74] Smart KM, Curley A, Blake C, Staines A, Doody C (2010). The reliability of clinical judgments and criteria associated with mechanisms-based classifications of pain in patients with low back pain disorders: a preliminary reliability study. J Man Manip Ther.

[75] Nijs J, Apeldoorn A, Hallegraeff H, Clark J, Smeets R, Malfliet A, et al. Low back pain: 2015 Guidelines for the clinical classification of predominant neuropathic, nociceptive, or central sensitization pain. Pain Physician. 2015;18:E333–46. 26000680

[76] Ford J, Story I, Sullivan P O, McKeenen J (2007). Classification systems for low back pain: a review of the methodology for development and validation. Phys Ther Rev.

[77] Dagenais S, Tricco AC, Haldeman S (2010). Synthesis of recommendations for the assessment and management of low back pain from recent clinical practice guidelines. Spine J.

[78] Serbic DPT (2014). Diagnostic uncertainty and recall bias in chronic low back pain. Pain.

[79] Barker ME, Fairbank JCT, Pynsent PB (1990). A practical classification of spinal pains based on a study of patients seen in British general practice over a five year period. Back pain: classification of syndromes.

[80] Nachemson AL, Andersson GB (1982). Classification of low back pain. Scand J Work Environ Health.

[81] Laslett M, van Wijmen P (1999). Low back and referred pain: diagnosis and a proposed new system of classification. New Zealand Journal of Physiotherapy.

