# Supplementary material for: Classification of patients with low back-related leg pain: a systematic review
Source: BMC Musculoskelet Disord. 2016 May 23;17:226. doi: 10.1186/s12891-016-1074-z (PMC4877814; doi:10.1186/s12891-016-1074-z)
Supplement: Additional file 1: — Medline Search Strategy. (DOCX 17 kb) [file 12891_2016_1074_MOESM1_ESM.docx]

**Additional File 1** Medline Search Strategy

| Database: Ovid MEDLINE(R) In-Process & Other Non-Indexed Citations and Ovid MEDLINE(R)  <1946 to Present> Date of Search: 26 july 2013 | |
| --- | --- |
| Back | 1     Back Pain/ (14558) 2     Spine/ (20674) 3     Back/ (3711) 4     lumbo$.ti,ab. (11604) 5     backache.ti,ab. (1956) 6     back ache.ti,ab. (52) 7     (spinal or spine).ti,ab. (247442) 8     lumbar.ab,ti. (73557) 9     "back pain".ab,ti. (28820) 10     Low Back Pain/ (14064) 11     1 or 2 or 3 or 4 or 5 or 6 or 7 or 8 or 9 or 10 (321052) |
| Leg | 12     (leg adj3 pain).ti,ab. (3559) 13     (nerve adj3 pain).ti,ab. (2193) 14     (radi$ adj3 pain).ti,ab. (6371) 15     neuropathic.ti,ab. (16915) 16     (referr$ adj3 pain).ti,ab. (2479) 17     "nerve root$".ti,ab. (8152) 18     Polyradiculopathy/ (2224) 19     Nerve Compression Syndromes/ (8997) 20     radicul$.ti,ab. (10340) 21     12 or 13 or 14 or 15 or 16 or 17 or 18 or 19 or 20 (52960) |
| Back and Leg | 22     11 and 21 (20495) |
| Sciatica | 23     Sciatica/ (4167) 24     sciatic$.ti,ab. (21982) 25     23 or 24 (23651) |
| Stenosis | 26     Spinal Stenosis/ (4152) 27     spinal stenosis.ti,ab. (2985) 28     26 or 27 (5250) |
| Disc | 29     Intervertebral Disc Displacement/ (15413) 30     ((disc or discs) adj1 (displacement$ or hernia$ or protru$ or avulsion$)).ti,ab. (6448) 31     ((disk or disks) adj1 (displacement$ or hernia$ or protru$ or avulsion$)).ti,ab. (2500) 32     29 or 30 or 31 (18525) |
| Non specific low back pain | 33     "non specific low back pain".ti,ab. (417) 34     "nonspecific low back pain".ti,ab. (323) 35     "low back-related leg pain".ti,ab. (16) 36     33 or 34 or 35 (744) |
| All back and leg pain | 37     22 or 25 or 28 or 32 or 36 (59153) |
| Classification | 38     Diagnosis/ (16563) 39     Diagnosis, Differential/ (372889) 40     (clinical adj1 predict$).ti,ab. (8801) 41     (clinical adj1 rule$).ti,ab. (186) 42     (predict$ adj3 (model$ or rule$)).ti,ab. (65237) 43     (diagnos$ adj3 (model$ or rule$)).ti,ab. (4638) 44     (classification or classified).ti,ab. (340737) 45     identification.ti,ab. (427912) 46     "subgroup$".ti,ab. (131764) 47     "sub-group$".ti,ab. (6528) 48     38 or 39 or 40 or 41 or 42 or 43 or 44 or 45 or 46 or 47 (1312408) |
| Classification of LBLP | 49     37 and 48 (5248) |
