# Supplementary material for: Classification of patients with low back-related leg pain: a systematic review
Source: BMC Musculoskelet Disord. 2016 May 23;17:226. doi: 10.1186/s12891-016-1074-z (PMC4877814; doi:10.1186/s12891-016-1074-z)
Supplement: Additional file 2: — Summary of the critical appraisal for all papers. (DOCX 23 kb) [file 12891_2016_1074_MOESM2_ESM.docx]

**Additional File 2** Summary of the critical appraisal for all papers (Y= yes; N= no; P=partial; DK=don’t know; NA= non applicable)

|  | Albert 2012 | Barker 1990 | Ben Debba 2000 | Bernard 1987 | Cassisi 1995 | Delitto 2012 | Fritz 2007 | Glassman 2011 | Hahne 2011 | Hall 1994 | Mckenzie 1981 | Nachemson 1982 | Nijs 2015 | Paatelma 2009 | Petersen 2003 | Roach 1997 | Schafer 2009 | Scholz 2009 | Spitzer 1987 | Smart 2011 | Sweetman 1992 | Vining 2013 |
| --- | --- | --- | --- | --- | --- | --- | --- | --- | --- | --- | --- | --- | --- | --- | --- | --- | --- | --- | --- | --- | --- | --- |
| **Purpose**  Is purpose, population and setting clearly specified? | Y | Y | Y | Y | Y | Y | Y | P | Y | P | Y | Y | Y | Y | Y | Y | Y | Y | Y | Y | Y | Y |
| **Content validity**  (i) Is the domain and all specific exclusions from the domain clearly specified? | Y | P | Y | P | Y | Y | Y | P | Y | P | Y | Y | Y | Y | Y | P | Y | Y | Y | Y | P | Y |
| (ii) Are all relevant categories included? | P | N | N | P | N | Y | N | N | N | Y | N | P | Y | Y | Y | P | Y | Y | Y | Y | P | Y |
| (iii) Is the breakdown of categories appropriate, considering the purpose? | Y | N | Y | Y | Y | Y | Y | P | Y | Y | Y | N | Y | Y | Y | DK | Y | Y | P | Y | P | Y |
| (iv) Are the categories mutually exclusive? | Y | N | Y | N | N | N | P | N | Y | Y | N | N | N | Y | N | N | P | Y | N | Y | N | N |
| (v) Was the method of development appropriate? | P | N | P | N | N | Y | Y | N | P | P | N | DK | P | N | P | N | P | Y | Y | Y | P | Y |
| (vi) If multiaxial, are criteria of content validity satisfied for each additional axis? | NA | NA | NA | NA | Y | NA | NA | NA | NA | NA | NA | NA | NA | NA | NA | NA | NA | NA | P | NA | NA | NA |
| **Face Validity**  (i) Is the nomenclature used to label the categories satisfactory? | Y | N | Y | Y | P | P | N | Y | Y | Y | Y | P | Y | Y | Y | Y | Y | Y | Y | Y | N | Y |
| (ii) Are the terms used based upon empirical (directly observable) evidence? | Y | N | Y | N | P | Y | P | Y | Y | Y | Y | Y | Y | P | Y | N | Y | Y | Y | Y | Y | Y |
| (iii) Are the criteria for determining inclusion into each category clearly specified? | Y | Y | Y | N | Y | P | Y | P | Y | Y | Y | Y | N | N | Y | P | Y | Y | Y | Y | Y | Y |
| (iv) If yes do these criteria appear reasonable? | Y | N | P | P | P | Y | Y | P | Y | P | Y | N | P | P | Y | P | Y | P | Y | P | N | Y |
| (v) Have the criteria been demonstrated to have reliability or validity? | Y | N | P | N | P | P | P | N | P | P | P | DK | P | DK | P | P | Y | P | P | P | N | P |
| (vi) Are the definitions of criteria clearly specified? | Y | P | Y | N | Y | P | Y | P | Y | Y | Y | Y | P | Y | P | P | Y | P | N | Y | N | Y |
| (vi) If multiaxial are criteria of face validity satisfied for each additional axis? | NA | NA | NA | NA | Y | NA | NA | N | NA | NA | NA | DK | NA | NA | NA | NA | NA | NA | N | NA | NA | NA |
| **Feasibility**  (i) Is the classification simple to understand? | Y | P | Y | N | Y | N | Y | P | P | Y | N | Y | Y | Y | Y | P | Y | Y | Y | Y | N | Y |
| (ii) Is the classification easy to perform? | Y | DK | Y | N | P | N | DK | Y | P | Y | N | Y | DK | Y | N | DK | Y | Y | P | Y | N | Y |
| (iv) Any special skills/tools or training required? | Y | N | P | Y | DK | N | P | DK | Y | Y | Y | N | Y | Y | Y | DK | Y | P | Y | Y | Y | P |
| (v) How long does it take to perform? | DK | DK | DK | DK | DK | DK | DK | DK | DK | DK | DK | DK | DK | 30m | 1hr | DK | DK | DK | DK | DK | DK | DK |
| **Construct Validity**  (i) Does it discriminate between entities thought to be different in a way appropriate for the purpose? | P | DK | P | DK | Y | Y | P | DK | N | Y | P | P | DK | DK | DK | DK | Y | Y | Y | Y | DK | DK |
| (ii) Does it perform satisfactorily compared to other systems classifying the same domain? | P | DK | DK | DK | DK | DK | DK | DK | N | DK | P | DK | DK | DK | P | DK | DK | Y | DK | DK | DK | DK |
| **Reliability**  (i) Does the system provide consistent results when classifying the same conditions? | DK | DK | DK | N | DK | P | DK | Y | DK | Y | Y | DK | DK | Y | DK | P | Y | DK | DK | Y | P | DK |
| (ii) Is the intraobserver and interobserver reliability satisfactory? | DK | DK | DK | N | DK | P | DK | Y | DK | Y | Y | DK | DK | Y | DK | P | P | DK | DK | Y | P | DK |
| **Generalisability**  (i) Has it been used in other studies &/or settings? | P | N | N | N | N | Y | N | N | N | Y | Y | Y | N | N | Y | N | Y | N | Y | N | N | P |
| **TOTAL OVERALL SCORE** | 4 | 2 | 3.5 | 2 | 3 | 3.5 | 3 | 2.5 | 3 | 5 | 5.5 | 3.5 | 2.5 | 3.5 | 4 | 3 | 5 | 4 | 4 | 5 | 2.5 | 3.5 |
